# Bee venom ameliorates gentamicin-induced kidney injury by restoring renal aquaporins and enhancing antioxidant and anti-inflammatory activities in rats

**DOI:** 10.3389/fphar.2025.1525529

**Published:** 2025-04-17

**Authors:** Doaa Abdelrahaman, Obeid Shanab, Ahmed Abdeen, Afaf Abdelkader, Khalid M. Elazab, Hagar Kouriem, Zainab M. Maher, Amal S. Abu-Almakarem, Mohamed E. Mohamed, Yasser M. Elbastawisy, Mohamed G. Elsehrawy, Abdelnaser A. Badawy, Naglaa Mokhtar, Moaz A. Mojaddidi, Madaniah O. Zakari, Samah F. Ibrahim, Dania Abdelhady, Laila Mostafa

**Affiliations:** ^1^ Department of Internal Medicine, College of Medicine, Princess Nourah bint Abdulrahman University, Riyadh, Saudi Arabia; ^2^ Department of Biochemistry, Faculty of Veterinary Medicine, South Valley University, Qena, Egypt; ^3^ Department of Forensic Medicine and Toxicology, Faculty of Veterinary Medicine, Benha University, Toukh, Egypt; ^4^ Department of Forensic Medicine and Clinical Toxicology, Faculty of Medicine, Benha University, Benha, Egypt; ^5^ Department of Biology, Faculty of Science, Jazan University, Jazan, Saudi Arabia; ^6^ Department of Pathology and Clinical Pathology, Faculty of Veterinary Medicine, South Valley University, Qena, Egypt; ^7^ Department of Basic Medical Sciences, Faculty of Applied Medical Sciences, Al-Baha University, Al-Baha, Saudi Arabia; ^8^ Department of Basic Medical Sciences, College of Medicine, AlMaarefa University, Riyadh, Saudi Arabia; ^9^ Department of Basic Medical Sciences, College of Medicine, Al-Rayan Colleges, Medina, Saudi Arabia; ^10^ Department of Human Anatomy and Embryology, Faculty of Medicine, Mansoura University, Mansoura, Egypt; ^11^ Department of Nursing Administration and Education, College of Nursing, Prince Sattam bin Abdulaziz University, Al-Kharj, Saudi Arabia; ^12^ Department of Nursing Administration, Faculty of Nursing, Port Said University, Port Said, Egypt; ^13^ Department of Medical Biochemistry, Faculty of Medicine, Northern Border University, Arar, Saudi Arabia; ^14^ Department of Medical Biochemistry, Faculty of Medicine, Mansoura University, Mansoura, Egypt; ^15^ Department of Basic Medical Sciences, College of Medicine, Taibah University, Medina, Saudi Arabia; ^16^ Department of Biomedical Sciences, Dubai Medical College for Girls, Dubai Medical University, Dubai, United Arab Emirates; ^17^ Department of Physiology, Faculty of Medicine, Benha University, Benha, Egypt

**Keywords:** ethnopharmacology, aminoglycoside, bee venom, aquaporins, oxidative stress, renal pharmacology

## Abstract

**Introduction:**

Gentamicin (GM) is a frequently used aminoglycoside for managing serious illnesses; nonetheless, renal complications limit its use. Bee venom (BV) is a biological toxin that exhibits anti-inflammatory and antioxidant activities. This study was designed to explore the mitigating effect of BV remediation on GM induced renal injury.

**Methods:**

Twenty male rats were divided into four groups (five rats each), namely, control (saline subcutaneously); BV group (1 mg/kg S/C twice weekly for 1 month); GM group (100 mg/kg i. p. for 1 week); and GM-BV group (the same aforementioned dosages of GM and BV, with GM administered in the last week for 4 weeks).

**Results and discussion:**

BV mitigated the GM-inflicted kidney damage, as evidenced by a substantial improvement in the renal function and oxidative state. In addition, a downregulation in the expression of inflammatory biomarkers (Casp-1, IL-6, TNF-α, and NF-κB/P65/P50) and an upregulation of oxidative stress marker expression (NRF2) were noticed. BV upregulated the expression of aquaporins (AQPs) and renal water channel proteins (AQP1 and AQP2), which are useful for the early detection of renal injury. Additionally, BV exposure exerted a mitigating effect on the apoptotic cascade, as evidenced by the downregulation of cleaved Caspase-3 (Casp-3) and cytochrome c (Cyto c). BV administration also led to an improvement in RBC, WBC, and platelet counts, along with enhanced Hb levels. Interestingly, BV could protect against GM triggered nephrotoxicity.

## 1 Introduction

Gentamicin (GM) is a well-known, affordable aminoglycoside, with low resistance and high efficacy against potentially lethal infections caused by gram-negative pathogens (Elgazzar et al., 2022). Notwithstanding its extensive therapeutic uses, it is widely recognized for producing considerable nephrotoxic consequences that seriously limit its use (Udupa and Prakash, 2019). The pathophysiology of GM-induced renal damage is multi-factorial. GM accumulates in the proximal convoluted tubules, resulting in glomerular congestion, tubular necrosis, and eventually renal failure (Abouzed et al., 2021). However, a key contributor to GM-mediated nephrotoxicity is the overabundance of deleterious free radicals and consequent oxidative harm (Abdeen et al., 2021). Reactive oxygen species (ROS), including hydrogen peroxide (H_2_O_2_), superoxide anion (O_2_^⋅–^), and hydroxyl radical (OH^⋅^), have been proven to be produced by GM, causing severe damage to various cellular molecules, including proteins, lipids, DNA, ultimately leading to apoptosis (Abdeen et al., 2021; Abdelnaby et al., 2022). Inflammatory processes are demonstrated to be a consequence of surplus ROS production (Ahmed et al., 2022). In addition, GM is assumed to affect the function of water channel proteins, specifically aquaporins (AQPs), in the kidney (Abdeen et al., 2014; Su et al., 2020).

AQPs are transmembrane protein channels that provide fluid translocation and modulate osmolarity and concentration of urine and fluid volume (Tamma et al., 2018). The proximal convoluted tubule serves as the prime site of absorption for the majority of fluids after they pass through glomeruli, where AQP1 is highly expressed (Abdeen et al., 2016; El-Agawy et al., 2022). In the kidney, AQP1 is crucial for tubule water permeability and countercurrent exchange processes (Candan et al., 2023). On the contrary, AQP2 is most abundant in the epithelial cells of collecting ducts, where it plays an indispensable function in controlling fluid volume and urine concentration (El-Agawy et al., 2022). According to certain reports, AQP expressions are strongly associated with acute kidney injury and are useful for the early diagnosis of such injury (Abdeen et al., 2014; Jiang et al., 2021). Accumulative evidence suggests that AQP1 and 2 undergo alterations in response to different insults such as puromycin (Abdeen et al., 2020), lipopolysaccharide (Candan et al., 2023), and methotrexate (El-Agawy et al., 2022).

Bee venom (BV, apitoxin), produced by bees (*Apis mellifera*), is one of the well-known naturally occurring beneficial biological toxins (Wehbe et al., 2019; Sadek et al., 2024). It is a combination of bioactive compounds with neurotoxic and immunogenic properties. It is composed of peptides including melittin, mast cell degranulation peptide, apamin, and adolapin and enzymes like phospholipase A2, acid phosphomonoesterase, hyaluronidase, and lysophospholipase; it additionally encompasses a variety of amines, including histamine, norepinephrine, dopamine, and volatile compounds (Kim et al., 2019). All bee products, including honey and BV, have been used for hundreds of years since religious books (the Bible and the Holy Quran) highlighted their medicinal benefits (Wehbe et al., 2019). BV remedy involves the medicinal use of bee stings or venom injection to alleviate a variety of illnesses and has been utilized in alternative medicine for more than 3000 years (Zhang et al., 2018). BV’s therapeutic potential is ascribed to its anti-inflammatory, antioxidant, antifibrotic, immunomodulatory, anticancer, and antiapoptotic properties (Kader et al., 2019; Eleiwa et al., 2023). Recent investigations have demonstrated that injecting BV may be beneficial in the alleviation of nephrotoxicity caused by agents such as cisplatin (Kim H. et al., 2020), acute endotoxic kidney injury (Kim et al., 2021), and acrylamide (Amra et al., 2018).

In consideration of the pharmacological properties of BV, we postulated that it could serve as a mitigating strategy for GM-induced toxicity. To the best of our knowledge, no research has been conducted to specifically investigate the potential effect of BV treatment on GM-triggered kidney injury. Therefore, the present research was conducted to assess the mitigating action of BV on GM-prompted kidney damage in rat models and evaluate and explore whether the AQP signaling pathway is associated with these impacts. Renal biomarkers, hematological profiles, oxidative stress indices, inflammatory and apoptotic marker expression, and histomorphological characteristics, were all assessed in this work.

## 2 Materials and methods

### 2.1 Chemicals and drugs

Lyophilized *A. mellifera* purified BV 1 mg/vial (Abevac^®^, VACSERA, Cairo, Egypt) was utilized. GM (Garamycin 80^®^; gentamicin sulfate 80 mg/2 mL vial) was obtained from Memphis Co. for Pharm. Chem. Ind., Cairo, Egypt.

### 2.2 Animals

Twenty male albino Wistar rats, weighing 120 ± 10 g, aged 8 weeks, were procured from the Centre for Laboratory Animals, Faculty of Veterinary Medicine, South Valley University, Egypt. Rats were raised for 2 weeks beforehand to the trial in well-aerated cages under standard temperature (22.5°C ± 2°C), relative humidity of 60% ± 10%, and light (12 h light/dark cycles). Throughout the study, rats were fed a conventional basal diet and given free access to water.

### 2.3 Experimental protocol

Following 2 weeks, rats were evenly divided into four groups (five rats each), namely, control group, where rats were given saline subcutaneously (S/C); BV group, where rats were injected BV S/C at a dose of 1 mg/kg twice weekly for 1 month, determined according to Kim et al. (2013); GM group, where the rats were i. p. injected 100 mg/kg of GM daily for 1 week based on our beforehand studies (Abdeen et al., 2021; Elgazzar et al., 2022); and GM-BV group, where both remedies were administered at the same aforementioned dosages (GM was given in the last week).

After 4 weeks, the experiment was halted, and blood was withdrawn from the retro-orbital venous plexus. The blood samples were divided into two portions; the first portion was collected in tubes containing EDTA to prevent clotting and used for hematological studies. The second portion was centrifuged at 5000 rpm for 10 min to obtain serum, which was then frozen at −20°C for subsequent biochemical analysis. Subsequently, all rats were anesthetized using 3%–4% isoflurane inhalation and euthanized by long exposure to anesthesia. Next, the kidneys were promptly retrieved and scrubbed with cold physiological saline to get rid of any congeals before being sliced. One portion was kept in 10% buffered neutral formalin for further histological inspection. Some tissue sections were kept at − 80°C for the extraction of RNA and proteins, while the remainder of fresh tissue pieces were kept at −20°C for an oxidative cascade biomarker investigation.

### 2.4 Assessment of renal function parameters and hematological profile

Serum levels of creatinine (catalog no. CR 1251), blood urea nitrogen (BUN; catalog no. UR 2110), and uric acid (catalog no. UA 2021) have been measured to assess kidney function. All proceedings were conducted in conformity with the manufacturer’s (Laboratory Bio Diagnostic Co., Giza, Egypt) protocols. An automated blood analyzer (URIT-2900 plus, URIT Medical Electronic Co., Shenzhen, China) was utilized to measure the red blood cells (RBCs), hemoglobin concentration (Hb%), white blood cells (WBCs), and platelet counts for the entire blood samples.

### 2.5 Preparation of tissue homogenates, antioxidants, and peroxidation biomarker assay

One gram of each tissue sample was homogenized using a sonicator homogenizer in an ice-cold buffer solution (K_3_PO_4_ 50 mmol, EDTA 1 mmol, pH 7.5). Next, we used a cooling centrifuge to spin the resulting homogenate at 4000 rpm for 20 min. The supernatant was gathered and stored at −80 °C for the measurement of glutathione peroxidase (GPx; catalog no. Gp2524) activity, total antioxidant capacity (TAC; catalog no. TA2513) content, and malondialdehyde (MDA; catalog no. MD2529) level using specialized kits from Laboratory Bio Diagnostic Co., Cairo, Egypt.

### 2.6 RNA seclusion with reverse transcription-PCR

Using the QIAzol Lysis Reagent (QIAzol^TM^, QIAGEN^®^, United States), total RNA has been extracted from the kidney homogenate in accordance with the manufacturer’s instructions. The total RNA content of the samples was assessed using a spectrophotometer (NanoDrop ND-1000 Spectrophotometer, Thermo Scientific, United States). The RNA quality was assessed using the absorbance ratio between 260 and 280 nm. The extracted total RNA was reverse transcribed into cDNA using the miScript II RT Kit (QIAGEN^®^, United States). In addition, 1 μg of RNA was converted to 1 μg of cDNA using an Oligo (dT) primer (PrimeScript^TM^, TaKaRa Bio Inc., CA, United States). Interleukin-6 (IL-6), tumor necrosis factor-α (TNF-α), nuclear factor erythroid 2-related factor 2 (NRF2), nuclear factor-κB/P65 (NF-κB/P65), superoxide dismutase type 1 (SOD1), caspase-1 (Casp-1), AQP1, AQP2, and kidney injury molecule-1 (KIM-1) were the primers used in the PCR that was conducted using a thermal cycler (A200 Gradient Thermal Cycler, LongGene^®^, Hangzhou, China) (Supplementary Table S1). A 1.5% agarose gel stained with ethidium bromide (Scientific Limited, Northampton, UK) in Tris-borate-EDTA buffer was used for electrophoretic separation of PCR products. The NIH ImageJ v1.47 program was employed to assess and recognize the band intensity in relation to the glyceraldehyde-3-phosphate dehydrogenase (GAPDH) gene. The bands were identified using a gel recording system (Bio-Rad, United States).

### 2.7 Western blotting

In accordance with the manufacturer’s instructions, the protein fraction was extracted from the organic phase of fatty tissue samples and treated with a proteinase inhibitor cocktail and phosphatase inhibitor tablet (Sigma-Aldrich, Germany, and PhosSTOP™, Roche Diagnostics, United States, respectively). Using SDS-polyacrylamide gel electrophoresis (SDS-PAGE), protein samples were loaded in equal amounts, extracted, and blotted onto a polyvinylidene difluoride membrane (Immobilon-P, Millipore). Primary antibodies (IL-6, TNF-α, NF-κB/P50, NF-κB/P65, NRF2, SOD1, cleaved Caspase-3 (C. Casp3–17/19), and cytochrome c (Cyto c), AQP1, AQP2, and KIM-1), which had been diluted, were used to probe the membranes following blocking in PBS-Tween (0.1%) with 1% BSA (Supplementary Table S2). The Roche Lumi-Light PLUS Kit and the Bio-Rad ChemiDoc Imaging System were used to recognize the bands. NIH ImageJ was used to quantify the intensities of the bands.

### 2.8 Histological inspection

The formalin-fixed renal sections were initially dehydrated by increasing alcohol concentration. Following that, xylene clearing was performed, followed by embedding in paraffin. The tissue specimens were cut into 5-µm sections and then stained with H&E for histopathological examination. A camera-integrated digital imaging system (DM300, Leica, Germany) was then used to scan the sections. For lesion scoring, three slides per animal were tested; thus, tubular injury was defined as tubular epithelial necrosis, cast formation, tubular dilatation, and the loss of the brush border, as described by Khalid et al. (2016), with minor modifications. The injury was scored by grading the percentage of affected tubules under 10 randomly selected, non-overlapping fields (magnification, × 200) as follows: 0, 0%; 1, ≤10%; 2, 11%–25%; 3, 26%–45%; 4, 46%–75%; and 5, 76%–100%. To score injured tubules, whole tubule numbers per field were considered the standard under a magnification of × 200. The injury score percentage was calculated in each field as follows: injury score (%) = number of injured tubules/number of whole tubules × 100.

### 2.9 Data analyses

Initially, all data were examined for homogeneity (Levene’s test) and normality (Shapiro–Wilk’s test). Then, all data were analyzed using a one-way analysis of variance (ANOVA), and the treatment means were compared using Duncan’s *post hoc* test (SPSS software, version 21; Inc., Chicago, IL, United States). Data are presented as the means ± SE. At *P* < 0.05, all results are deemed statistically significant with a 95% confidence interval. Data visualization was conducted using OriginPro (version 2019b). After data transformation, principal component analysis (PCA) was performed using the ‘factoextra’ and the ‘FactoMineR’ packages which were built in RStudio under R version 4.0.2.

## 3 Results

### 3.1 Biochemical parameter evaluation

**FIGURE 1:**
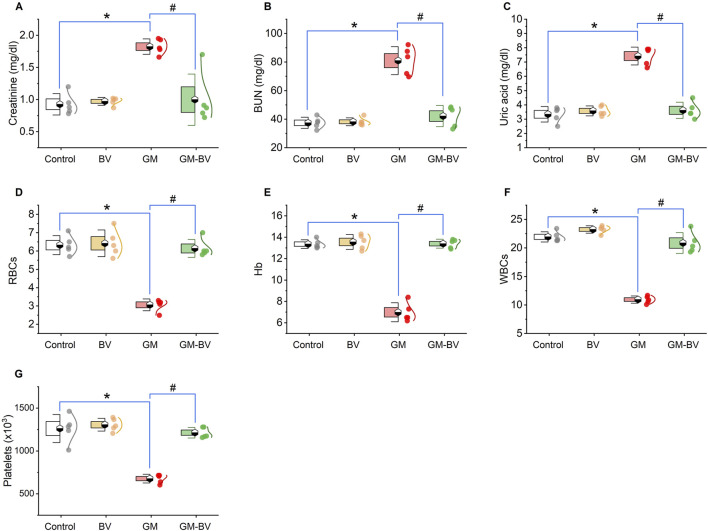
Dot-box plot of kidney functions and hematological parameters of GM-exposed rats upon BV remediation. **(A)** Creatinine; **(B)** BUN, blood urea nitrogen; **(C)** uric acid; **(D)** RBCs; **(E)** Hb, hemoglobin; **(F)** WBCs; **(G)** platelets. BV, bee venom; GM, gentamicin. Values were expressed as the mean ± SE (n = 5). *P* < 0.05; *GM vs*.* Control; ^#^GM-BV vs*.* GM.

As displayed in Figures 1A–C, GM injection prompted renal damage, as evidenced by a notable (*P* < 0.05) increase in renal function test markers (creatinine, BUN, and uric acid levels) compared to control rats. Conversely, treatment with BV remedy robustly (*P* < 0.05) decreased the GM-induced injuries in renal tissues, as exhibited by a noteworthy improvement in kidney function compared to GM-intoxicated rats.

### 3.2 Hematology profile

Hb%, RBCs, WBCs, and platelet counts are depicted in Figures 1D–G, respectively. Significant (*P* < 0.05) decreases in all measured hematological parameters following GM insult were detected. Interestingly, when GM was used concurrently with BV, the blood profile was retrieved nearly to normal compared to GM-treated rats.

### 3.3 Antioxidants and lipid peroxidation indices

**FIGURE 2:**
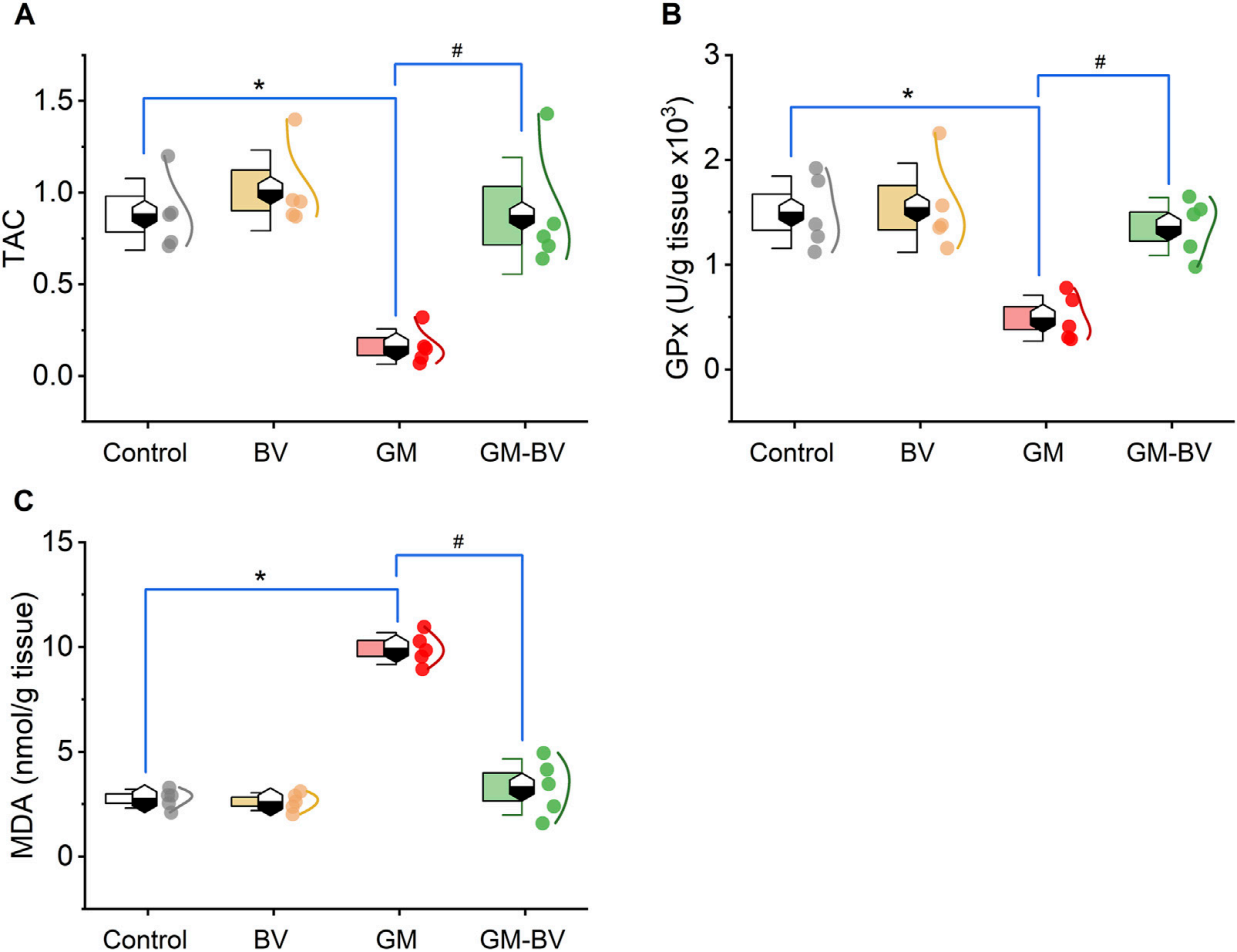
Dot-box plot of renal oxidative stress markers of GM-exposed rats upon BV remediation. **(A)** TAC, total antioxidant capacity; **(B)** GPx, glutathione peroxidase; and **(C)** MDA, malondialdehyde. BV, bee venom; GM, gentamicin. Values were expressed as the mean ± SE (n = 5). *P* < 0.05; *GM vs*.* Control; ^#^GM-BV vs*.* GM.

**FIGURE 3:**
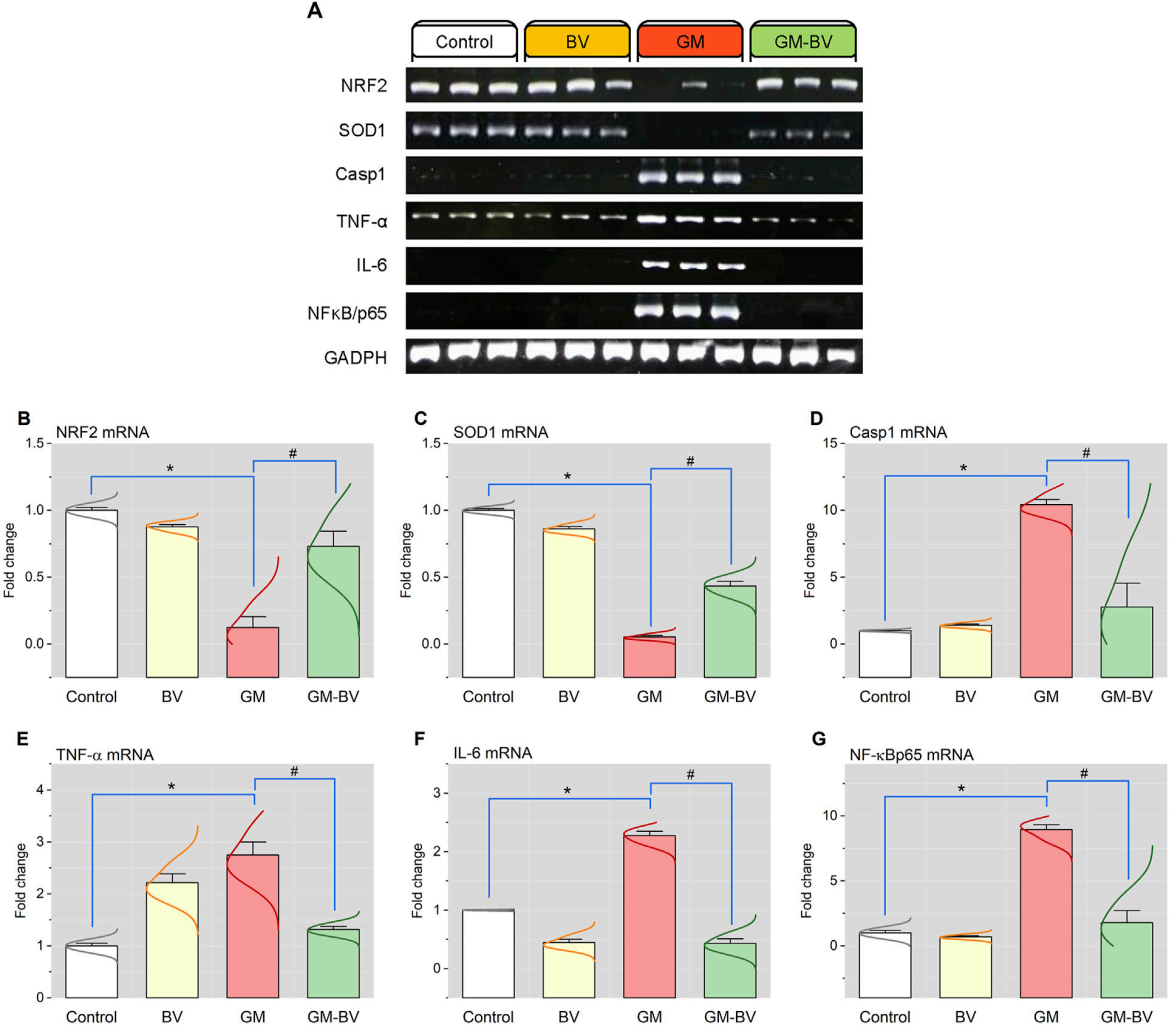
Renal mRNA expression of antioxidant and inflammation-related genes of GM-exposed rats upon BV remediation. **(A)** Representative bands of NRF2, SOD1, Casp-1, TNF-α, IL-6, NF-κB/P65, and GAPDH genes. Bar-plot panel of the semiquantitative analysis of NRF2, nuclear factor erythroid 2-related factor **(B)**; SOD1, superoxide dismutase **(C)**; Casp-1, caspase-1 **(D)**; TNF-α, tumor necrosis factor-α; (E) IL-6, interleukin 6 **(F)**; and NF-κB/P65, nuclear factor kappa-B transcription factor/P65 **(G)**. BV, bee venom; GM, gentamicin. Values were expressed as the mean ± SE (n = 5). P < 0.05; *GM vs. Control; #GM-BV vs. GM.

**FIGURE 4:**
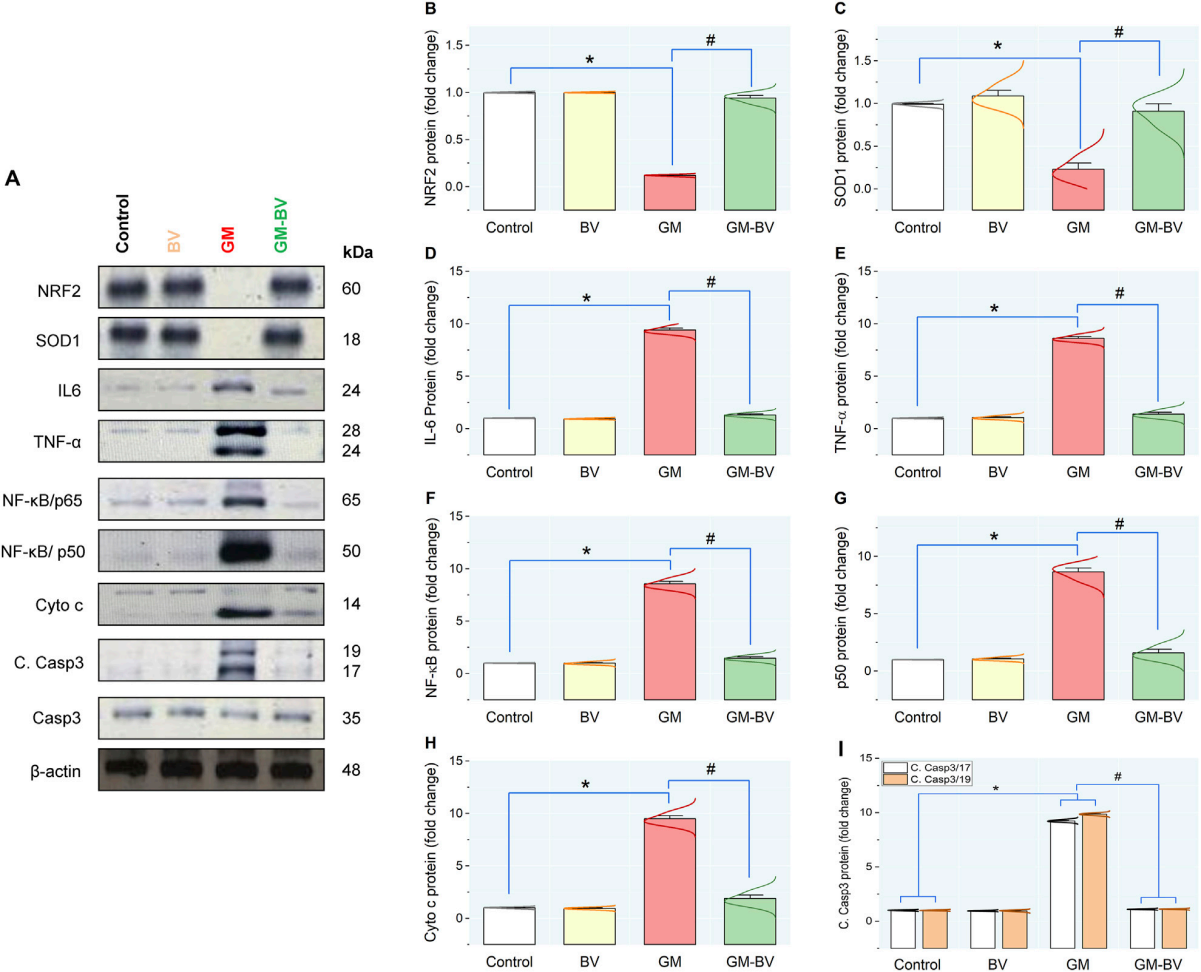
Protein expression levels of pro-inflammatory cytokine, antioxidant, and apoptotic markers in the kidney of GM-exposed rats upon BV remediation. **(A)** typical immunoblots of NRF2, SOD1, IL-6, TNF-α, NF-κB/P65, NF-κB/P50, Cyto c, C. Casp3 -17/19, Casp-3, and β-actin proteins. Bar-plot panels were created from immunoblot by semi-quantitative analysis for NRF2, nuclear factor erythroid 2-related factor **(B)**; SOD1, superoxide dismutase **(C)**; IL-6, interleukin-6 **(D)**; TNF-α, tumor necrosis factor α **(E)**; NF-κB/P65, nuclear factor kappa-B transcription factor/P65 **(F)**; NF-κB/P50, nuclear factor kappa-B transcription factor/P50 **(G)**; Cyto C, Cytochrome C **(H)**; and C. Casp3 -17/19, Cleaved Caspase-3 **(I)**. BV, bee venom; GM, gentamicin. Values were expressed as the mean ± SE (n = 5). P < 0.05; *GM vs. Control; #GM-BV vs. GM.

Data on antioxidant enzyme activities (GPx), TAC content, and MDA levels are displayed in Figure 2. MDA levels were substantially (*P* < 0.05) increased together with a noticeable (*P* < 0.05) reduction in TAC content and GPx activity upon GM intoxication. Additionally, the expression (mRNAs and proteins) levels of NRF2 and SOD1 in kidney tissue displayed notable (*P* < 0.05) downregulation, corroborating the instigation of oxidative stress (Figures 3, 4). Remarkably, GM-triggered oxidative damage was considerably (*P* < 0.05) hampered when GM was injected in conjunction with BV compared to GM-exposed rats.

### 3.4 Pro-inflammatory cytokine expression

As shown in Figures 3, 4, GM treatment provoked kidney tissue inflammation, as indicated by enhanced (*P* < 0.05) upregulation of mRNAs (Casp-1, TNF-α, IL-6, and NF-κB/P65) and protein expression (NF-κB/P65, NF-κB/P50, TNF-α, and IL-6) of inflammatory markers in contrast to controls. Interestingly, when BV was administered to GM-exposed rats, the GM harmful effect was mitigated, as evidenced by the modulation of the mRNA and protein expression levels of pro-inflammatory cytokines.

### 3.5 Evaluation of apoptotic marker expression in the kidney

As illustrated in Figure 4, GM intoxication substantially stimulated apoptotic cell death in renal tissue, as indicated by a noteworthy (*P* < 0.05) upregulation of protein expression levels of C. Casp3-17/19, along with an increase in Cyto c, compared to controls, suggests the promotion of apoptotic cell death. Conversely, synchronous BV administration considerably suppresses GM-induced apoptosis by modulating the expression of these apoptotic biomarkers in the kidney.

### 3.6 mRNA and protein expressions of AQP1, AQP2, and KIM-1

**FIGURE 5:**
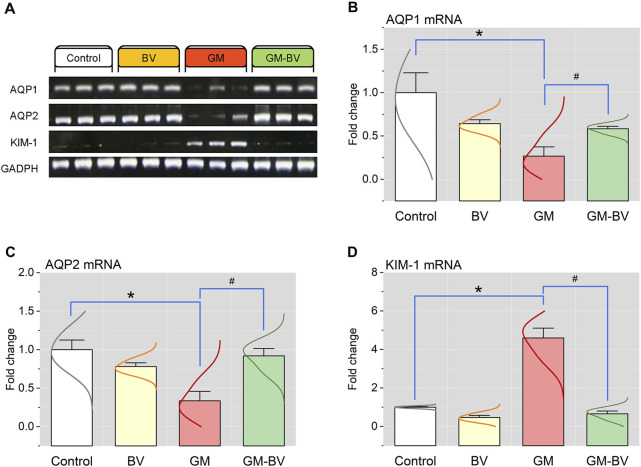
Renal mRNA expression of AQP1, AQP2, and KIM-1 genes of GM-exposed rats upon BV remediation. **(A)** Representative bands of AQP1, AQP2, KIM-1, and GAPDH genes. Bar-plot panels of mRNA expression levels of AQP1, aquaporin 1 **(B)**; AQP2, aquaporin 2 **(C)**; and KIM-1, kidney injury molecules 1 **(D)**. BV, bee venom; GM, gentamicin. Values were expressed as the mean ± SE (n = 5). P < 0.05; *GM vs. Control; #GM-BV vs. GM.

**FIGURE 6:**
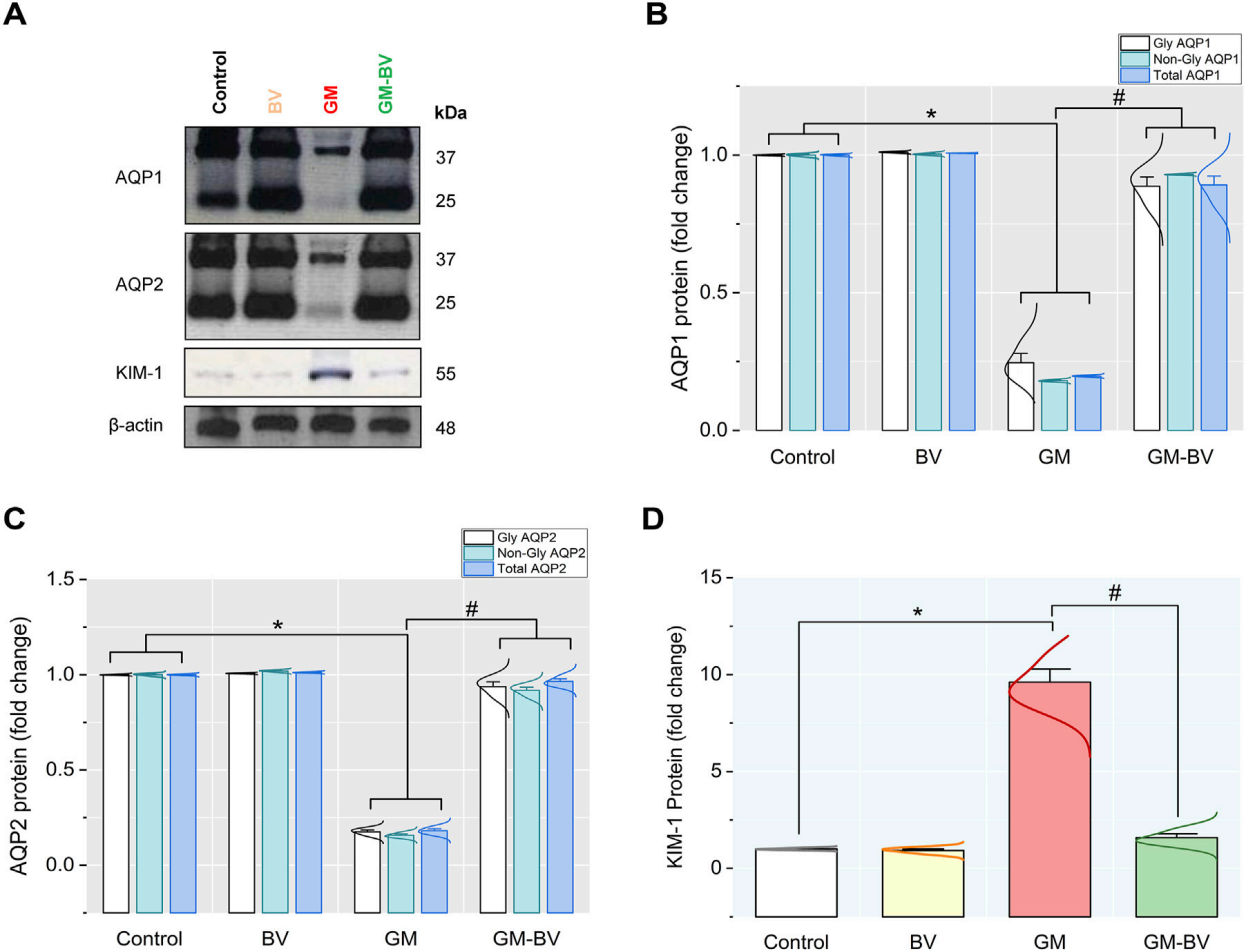
Protein expression levels of AQP1, AQP2, and KIM-1 in the kidney of GM-exposed rats upon BV remediation **(A)** AQP1, AQP2, KIM-1, and β-actin protein typical immunoblots. Bar-plot panels were created from immunoblot by semi-quantitative analysis for **(B)** glycosylated and non-glycosylated AQP1, aquaporin 1; **(C)** glycosylated and non-glycosylated AQP2, aquaporin 2; and **(D)** KIM-1, kidney injury molecule-1. BV, bee venom; GM, gentamicin. Values were expressed as the mean ± SE (n = 5). *P* < 0.05; *GM vs*.* Control; ^#^GM-BV vs*.* GM.

To demonstrate the nephroprotective effects of BV after GM exposure, renal expressions of AQP1, AQP2, and KIM-1 were investigated. AQP1, AQP2, and KIM-1 mRNA and protein expression are displayed in Figures 5, 6, respectively. Both AQPs were downregulated along with an increased expression of KIM-1 after GM intoxication. As depicted in Figure 6A, immunoblotting revealed the presence of two bands, namely, non-glycosylated AQP1/25 and glycosylated AQP1/37, both of which were considerably (*P* < 0.05) reduced in the GM-exposed group. Conversely, BV administration significantly (*P* < 0.05) reduced GM-induced kidney damage, as elucidated by increased levels of protein expression AQPs and KIM-1.

### 3.7 Multivariate analyses

**FIGURE 7:**
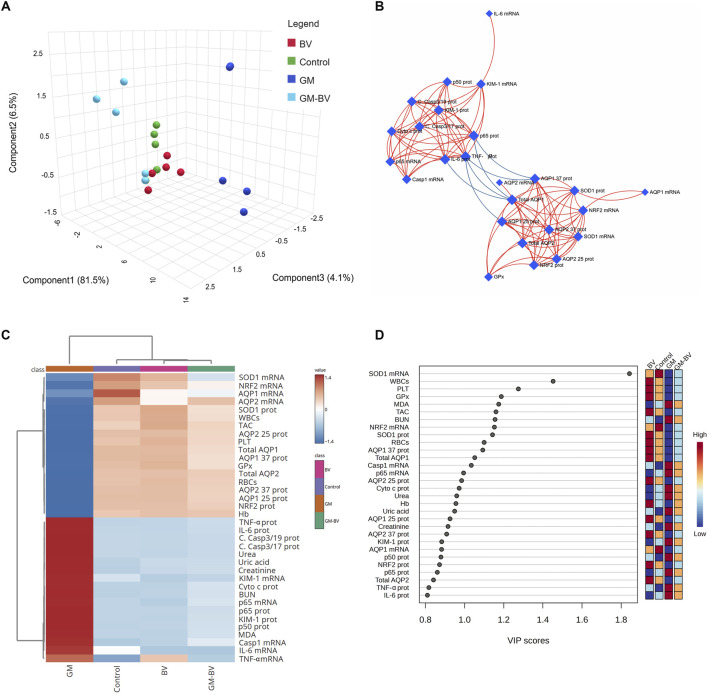
Clustering analysis of entire datasets in the renal tissue of GM-exposed mice upon BV remediation Pearson’s rank correlation coefficient. **(A)** Three-dimensional plot of PCA for identifying the all-experimental groups. **(B)** Correlation network. The network’s nodes display the measured variables, while its edges reflect the correlation measures. The correlation’s strength is proportionate to the color of the line. The blue lines display a negative correlation, while the red lines display a positive correlation. **(C)** Clustering heatmap. The rows and columns of the map are made up of various averages and treatment groups, respectively, and each colored cell on the map indicates the concentration value. On the grading scale, blue has the lowest value and red has the highest value. **(D)** VIP scores; the extent to which different factors contribute. The gradation scaling has the lowest value at the bottom and the highest value at the top. BV, bee venom; GM, gentamicin; PCA, principal component analysis; VIP, variable importance in projection.

Multivariate analyses were implemented to ascertain the correlation between all measured variables and treated groups, as depicted in Figure 7. PCA was used to investigate the association among different treatments and covariates (Figure 7A). The PCA distinguished three main dimensional components for all variables, which collectively represented 92.1% of the variation. Component 1 accounted for most of the examined variables and expressed the largest percentage of variation (81.5%), whereas components 2 and 3 exhibited smaller proportions of variance (6.5% and 4.1%, respectively). The PCA divulged that the GM-intoxicated group was obviously distinct from all other groups. The control, BV, and GM-BV groups were grouped on the left side with each other and reclused from the GM-exposed one. These findings elucidated a significant distinction between the intoxicated animals with GM- and GM-BV-treated groups. Additionally, correlation networks are constructed among variables in the control and exposed groups (Figure 7B). AQP1 and AQP2 protein expression were positively correlated with each other, as well as with SOD1, GPx, and NRF2, while they were negatively correlated with NF-κB/P65, NF-κB/P50, TNF-α, and IL-6 protein expression. Furthermore, the clustering heatmap was conducted to provide an intuitive visualization of all the data sets (Figure 7C). It reveals the noticeable distinction between the concentration levels of all variables in relation to various treatments. These data explicate how the GM-intoxicated rats displayed more injuries than rats in the other groups. Ultimately, the variable importance in projection (VIP) score, depicted in Figure 7D, showed that SOD1 mRNA expression, WBC count, platelet count, GPx, MDA, TAC, BUN, NRF2 mRNA expression, and SOD1 protein expression were the most significant variables distinguishing GM-exposed rats from the other groups.

### 3.8 Kidney histoarchitecture inspection

**FIGURE 8:**
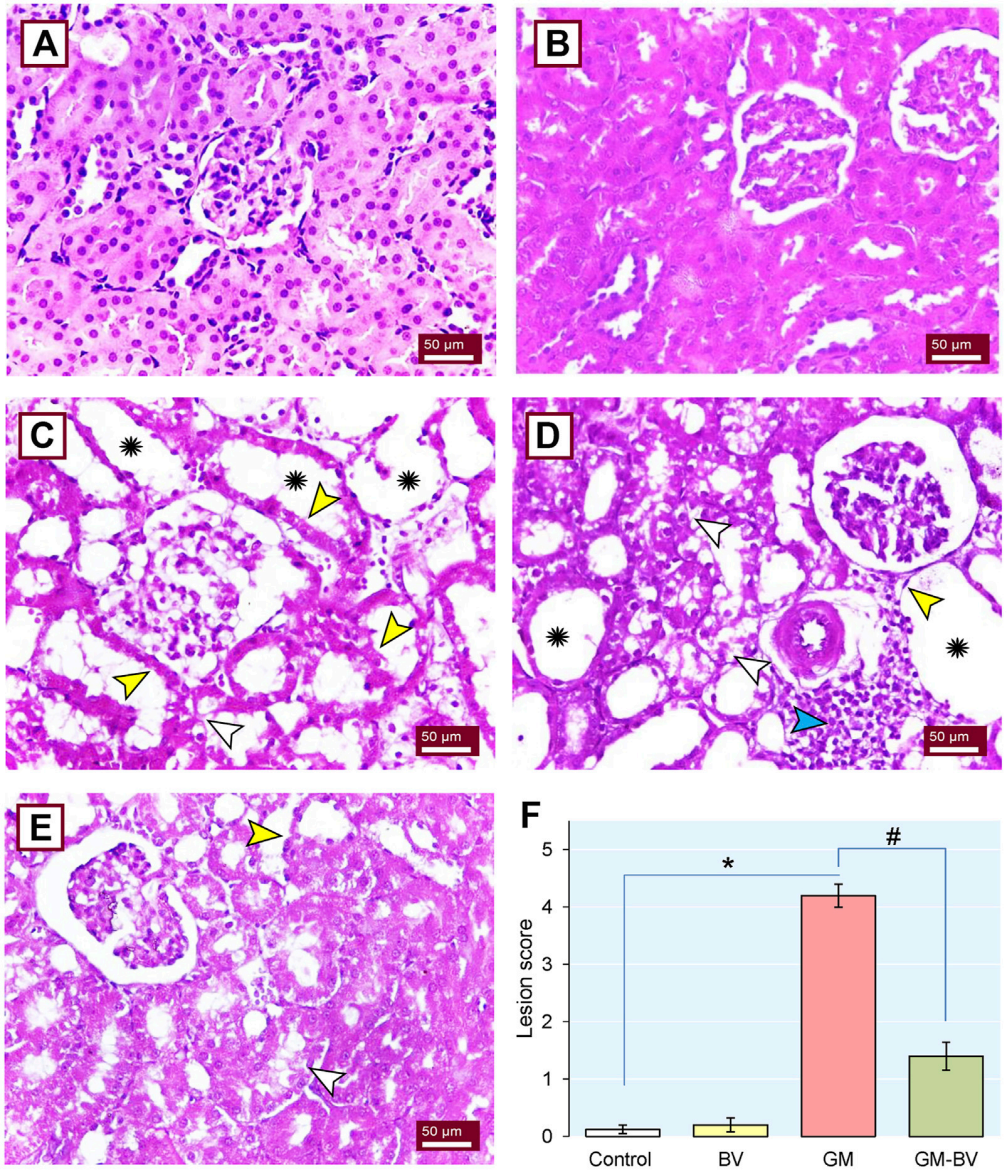
Histoarchitectural changes in the kidney tissue of GM-exposed rats upon BV remediation. Normal nephrons were observed in control **(A)** and BV-treated **(B)** rats. **(C and D)** Kidney section of the GM-treated group displayed loss of brush border (yellow arrowhead), vacuolated cytoplasm in the epithelial lining of renal tubules (white arrowhead), dense inflammatory cell infiltration (blue arrowhead), and severe tubular dilation (asterisk). **(E)** Dramatic improvement in renal architecture was observed when GM was co-administered with BV (GM-BV group), as indicated by regenerated glomeruli, renal tubular epithelium, decreased cystic space in renal tubules, and mild lesions in certain renal tubules. **(F)** Quantitative lesion scoring; values were expressed as the mean ± SE (n = 5). *P* < 0.05; *GM vs*.* Control; ^#^GM-BV vs*.* GM. (H&E ×400; scale bar = 50 µm).

The architectural perturbations in the kidney tissues of GM-intoxicated rats following BV supplementation was assessed to validate the previously reported observations (Figure 8). The control (Figure 8A) and BV (Figure 8B) groups exhibited normal patterns of histological architecture of the renal parenchyma. Nevertheless, the GM-exposed rats displayed vacuolar degeneration, renal tubular desquamation, cystic dilatation of renal tubules, tubular necrosis, interstitial edema, and prominent inflammatory cell transudation (Figures 8C, D). As depicted in Figure 8E, the GM-BV group expounded signs of regenerated glomeruli, renal tubular epithelium, and decreased cystic space in renal tubules. The lesion scoring presented in Figure 8F confirmed the significant damage occurred by GM intoxication and the protective effect of BV against GM-induced damage.

## 4 Discussion

The present data revealed considerable oxidative damage following GM treatment, as expounded by a substantial reduction in the GPx activity and TAC concentration along with increased MDA levels in kidney tissues. Furthermore, there was a downregulation of NRF2 and SOD1 protein and gene expressions. These results align with previous research (Yarijani et al., 2019; Abouzed et al., 2021; Nadeem et al., 2023). The transcription factor NRF2 is a crucial modulator of the cellular detoxification mechanism, which boosts the expression of antioxidant enzymes, such GPx and SOD (Habotta et al., 2023). Astonishingly, GPx is an intrinsic antioxidant enzyme that functions as the first line of antioxidant enzymatic safeguard crucial for breaking down the produced H_2_O_2_ into H_2_O and O_2_ (Ghaznavi et al., 2016; Ahmed et al., 2022). Alternatively, when this enzyme was exhausted as reported in our GM-intoxicated group, OH^⋅^ was produced in substantial quantities by Fenton’s reaction. OH^⋅^ is the primary regulator of the cell membrane’s lipid peroxidation (LPO) that generates MDA in the tissues (Abouzed et al., 2021). Even worse, MDA can interact with or crosslink DNA and proteins, causing adduct formation and biomolecular damage (Petrovic et al., 2020; Ahmed et al., 2022). Consistent with this assertion, our investigation revealed a substantial increase in blood levels of renal biomarkers (creatinine, BUN, and uric acid), concurrent with LPO in the cortical tubular brush border in renal histopathology following GM exposure, indicating severe deterioration in kidney function. Intriguingly, we found GM intoxication associated with the downregulation of AQP1 expression in renal tissue. The descending thin limb and proximal tubule exhibit noteworthy levels of AQP1 expression, which facilitates the transport O_2_ to extracellular space, thereby reducing intracellular ROS generation and mitochondrial damage to prevent cellular harm (Liu et al., 2020; El-Agawy et al., 2022). These findings coincide with the results of Lee et al. (2001), who reported a considerable decline in AQP1 expression in the cortex following the GM treatment, which may explain the disruption of the proximal tubules. Furthermore, in the present study, GM-intoxicated rats exhibited a notable increase in kidney tubular damage biomarkers, including KIM-1, a transmembrane glycoprotein that is notably overexpressed in proximal convoluted tubule epithelial lining during kidney injury (Alomair et al., 2023). GM is excreted unaltered through glomerular filtration and accumulates in the endosomes of the proximal convoluted tubule, leading to the disruption of the brush border and the release of GM into the cytosol. The released GM triggers free radical production through its effects on the endoplasmic reticulum and mitochondria, causing tubular necrosis and ultimately leading to renal failure (Yarijani et al., 2019; Abouzed et al., 2021). On the other hand, GM stimulates the release of iron from the mitochondria of the renal cortex, forming an iron–GM complex that promotes further free radical formation and enhances ROS generation (Ghaznavi et al., 2016; Abouzed et al., 2021). These findings corroborate our previous research, indicating that the proximal tubule is the prime target of GM-triggered kidney damage. When the proximal tubule is damaged, tubular reabsorption is impaired, leading to increased blood levels of urea and creatinine (Abdeen et al., 2021). Additionally, Alomair et al. (2023) reported that damage to the proximal tubules inhibited the clearance of waste products, thereby elevating their levels in the serum. In addition to impairing proximal tubule function, GM has also been implicated in collecting duct dysfunction, as evidenced by a reduction in the expression level of AQP2 in the current study. AQP2 is a water channel protein distributed to the cells of the renal collecting duct (Su et al., 2020). Along with proximal tubule cells, these findings are further corroborated by the detection of GM in collecting duct and distal tubule cells (Abdeen et al., 2014).

The oxidative stress and increased ROS production are incriminated in the initiation of renal inflammatory response in GM-intoxicated rats (Abdeen et al., 2021). A growing body of literature reveals a substantial correlation between oxidative stress and inflammation (Ahmed et al., 2022). Enhanced ROS formation is speculated to promote pro-inflammatory gene expression and the migration of inflammatory cytokines, thereby triggering an inflammatory response (Habotta et al., 2023). Consequently, we postulated that the inflammatory response is an additional potential pathway embroiled in GM-provoked renal injury (Nadeem et al., 2023). The inflammatory status encountered in the present investigation was detected in the histological findings in GM-treated rats, which was further substantiated by the notable increase in the expression of Casp-1 and the inflammatory cytokines, including TNF-α, IL-6, and NF-κB/P65/P50. Casp-1 is a widely recognized inflammation-mediated caspase that is crucial for the modulation of pro-inflammatory cytokines. Once matured, the cytokines trigger further signaling cascades that result in the induction of an inflammatory response (Molla et al., 2020). In the cytosol, the transcription factor NF-κB forms a complex with the inhibitory protein IκBα. Pro-inflammatory stimuli cause IκB to undergo rapid phosphorylation and degradation, releasing the P65/P50 heterodimers and enabling their translocation to the nucleus, where they activate NF-κB target genes (Li et al., 2019). Recent research indicates that the activation of NF-κB in epithelial cells of the renal tubule worsens tubular damage and escalates kidney inflammation (Shanab et al., 2023). TNF-α is the most paramount inflammatory mediator entangled in the pathogenesis of inflammatory response. Proinflammatory cytokines, including IL-6, are stimulated by the TNF-α and NF-κB/P65 pathways, together with adhesion molecules that encourage the peregrination of leukocytes to the inflammatory zone (Ahmed et al., 2022). Consistent with our results, Yarijani et al. (2019) found that GM exposure led to leukocyte seepage, cytokine release, and the activation of TNF-α and NF-κB. Furthermore, the disruption of AQP1 and AQP2’s protein and gene expression in the renal membrane also plays a role in the inflammatory response in our study, as elucidated by our correlation network. Many investigations have demonstrated that the downregulation of AQP expression is a primary pathological pathway of inflammatory damage through impairing the suppression of inflammation. AQP1 is crucial in the modulation of inflammation through the inhibition of inflammatory cell leakage, inflammatory factor secretion, and pseudopodium formation (Liu et al., 2020). Protein expression of AQP1 downregulated TNF-α, and AQP2 expression was decreased via the NF-κB pathway. Consequently, it serves as a sensitive biomarker for inflammation (Li et al., 2019).

Hematological profiles are commonly correlated with a patient’s health and play a crucial diagnostic role in clinical evaluation (Elyazji and Abdel-Aziz, 2013; Kuatsienu et al., 2017). In this study, the injection of GM significantly disrupts the hematological parameters. GM-induced anemia may be ascribed to the decreased erythropoietin hormone and enhanced RBC fragility following GM treatment. The erythropoietin suppression in the renal cortex modulated the hematological indices in rats, which is the humoral regulator of RBC production (Zubairu et al., 2021). Furthermore, the current uremia impeded the erythropoiesis and resulted in intravascular hemolysis (Arafat et al., 2017). Similarly, Jannat et al. (2018) demonstrated that exposure to GM affects bone marrow hematopoietic cells. In addition, Elyazji and Abdel-Aziz (2013) reported a decrease in Hb and WBC count in rabbits injected with GM. Moreover, Zubairu et al. (2021) reported that GM exposure caused a decrease in platelet count in rats.

A noteworthy cytotoxic effect of GM is the induction of apoptosis, as demonstrated in mesangial cells and proximal tubules. The mitochondrial pathway is well-known for its involvement in GM-induced apoptosis, with ROS proposed as the key mediators of this mechanism (Ahn et al., 2012). GM promotes the apoptotic pathway in renal tubules, triggers necrosis of the cell, disrupts the respiratory chain, and enhances the production of ROS (Yarijani et al., 2019). Irreversible damage to the mitochondrial membrane results in the unleashing of Cyto c into the cytoplasm; consequently, Casp-3 is activated, commencing the apoptotic cascade (Shanab et al., 2023). Accordingly, this study found that the renal tissue dramatically overexpressed the apoptotic biomarkers (C. Casp3-17/19 and Cyto c). Therefore, in conjunction with previous studies, our results obviously imply that apoptotic pathways are crucial in GM-induced kidney damage (Abouzed et al., 2021; Elgazzar et al., 2022). Intriguingly, according to reports, AQP expressions are correlated with apoptosis, which is corroborated by our correlation network. AQP1 is positively correlated with Casp-3 expression and activity (Gao et al., 2016).

BV has been demonstrated to exhibit different biological activities, such as anti-inflammatory and antioxidant properties. Thus, BV remedy has long been used as a conventional medicine for a variety of illnesses (Kim J.-Y. et al., 2020). It is highly regarded for its potent antioxidant properties, which are attributed to its melittin content as melittin is the main component of BV extracts (Somwongin et al., 2018; Mohamed et al., 2019). However, some reports suggest that melittin alone has very weak antioxidant activity compared to BV extract, which could be due to the presence of other components in BV extracts (Martinello and Mutinelli, 2021). These components include antioxidant enzymes from the honeybee venom gland tissue’s proteome, such as SOD1, peroxiredoxin 2540, and thioredoxin peroxidase1 isoform A (Peiren et al., 2008). There is copious evidence corroborating the antioxidant property of BV through increasing levels of antioxidant activity, along with mitigation of LPO (Hanafi et al., 2018; Eleiwa et al., 2023). Nearly, 18 active pharmacological components, including sulfur, peptides, enzymes, and amines, are present in BV (Ali, 2012). Among these, melittin is particularly renowned for its significant anti-inflammatory properties (Mohamed et al., 2019; Bava et al., 2023). In addition, sulfur is considered the primary constitute of BV, prompting the secretion of cortisol, which functions as an anti-inflammatory hormone (Ali, 2012). BV exerts its anti-inflammatory effects by blocking primary signaling pathways (including NF-κB protein), resulting in the inhibition of inflammatory cytokine release (IL-6 and TNF-α) and reducing tissue inflammation (Wehbe et al., 2019). Furthermore, BV injection has showed an antiapoptotic effect by downregulating Bax and Casp-3 levels (Mohamed et al., 2019). Consequently, the ongoing study elucidates that treatment with BV attenuated GM-induced renal damage, as seen by remarkable betterment in kidney functions, oxidative/antioxidative state, AQPs expression, and inflammatory biomarker levels. In addition, we noted an improvement of histological changes following BV injection, corroborating its mitigating effects. Our findings align with previous research that elucidated the antioxidative role of BV injection in a lipopolysaccharide-induced acute renal damage (Kim J.-Y. et al., 2020). In addition, our study displayed a significant increase in measured blood parameters following BV administration, which may be an indication of erythropoiesis stimulation (Abo-Zaid et al., 2023). These results are consistent with the findings of Mohammed and Hassan (2019), who recorded a noteworthy increase in the values of Hb in the BV-treated arthritis group. In addition, Son et al. (2007) reported that BV can enhance circulation and increase blood flow in the microvessels and play a role in promoting erythrocyte production.

**FIGURE 9:**
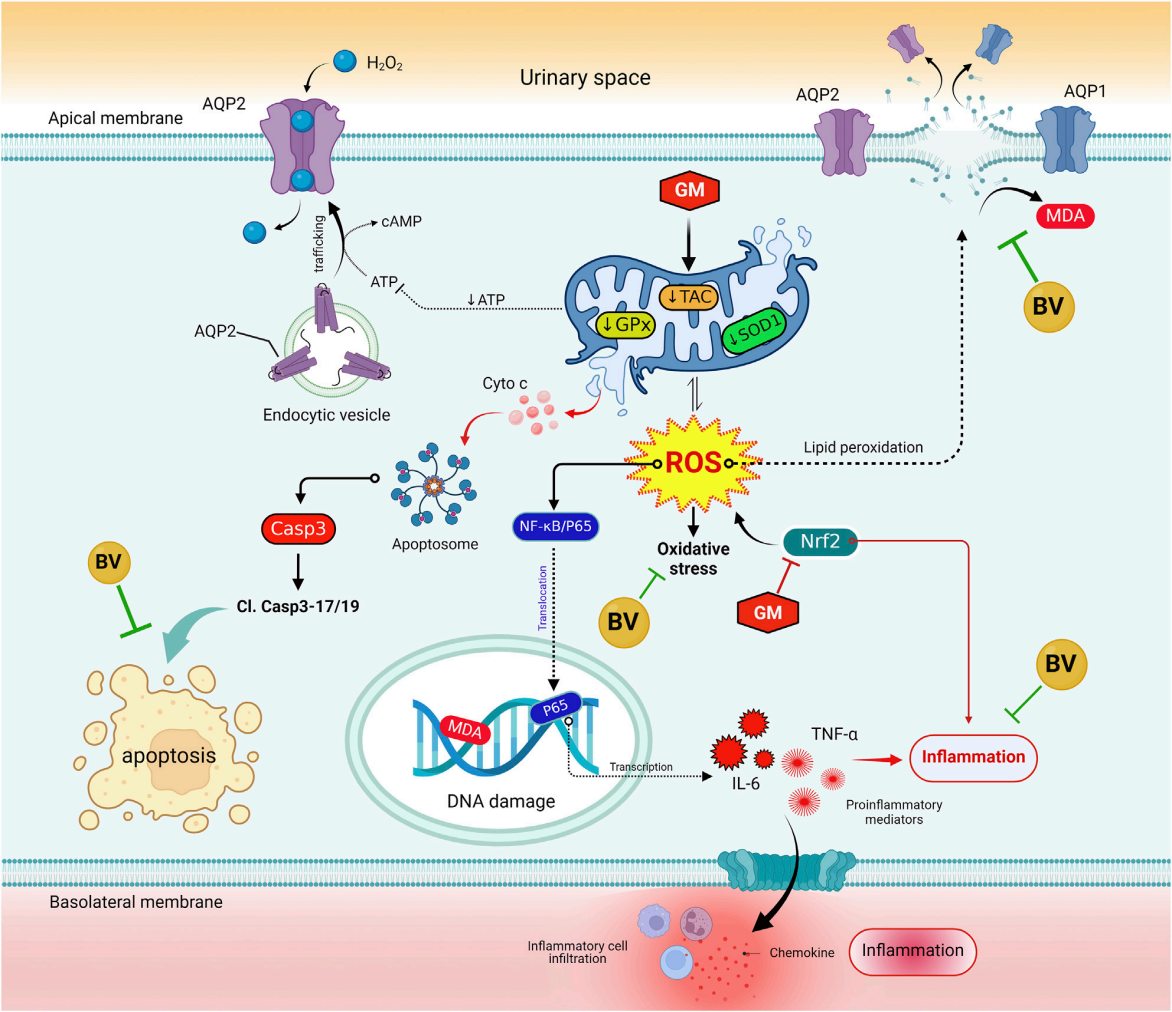
Molecular mechanisms that bolster the preemptive prospect of BV against GM-instigated nephrotoxicity. AQP1, aquaporin 1; AQP2, aquaporin 2; BV, bee venom; Cl. Casp-3; cleaved caspase-3; Casp-1, caspase-1; GM, gentamicin; GPx, glutathione peroxidase; IL-6, interleukin 6; KIM-1, kidney injury molecules 1; MDA, malondialdehyde; NF-κB/P65, nuclear factor kappa-B transcription factor/P65; NRF2, nuclear factor erythroid 2-related factor; ROS, reactive oxygen species; SOD1, superoxide dismutase; TAC, total antioxidant capacity; TNF-α, tumor necrosis factor-α.

In addition to the conventional single-variate analysis, we evaluated the effects of BV and GM interventions on the kidney tissue of rats using multivariate statistical analyses. The PCA results revealed that GM toxicity promotes a detrimental alteration in all measured variables, enabling these animals to be clearly discriminated against the healthy rats. The clustering heatmap highlighted discernible differences in all parameter concentrations compared to GM-intoxicated groups and other experimental groups. Therefore, these findings remarkably corroborate the potential mitigating impact of BV against GM toxicity. A significant proportion of the variation among treated groups was noticed to be explained by SOD1 mRNA expression, platelet count, WBC count, MDA, AQP1 protein expression, NRF2 mRNA, and RBC count, which is deemed a novel exploration, as delineated by VIP scoring data. Clustering analysis is, therefore, a crucial approach to comprehending the mechanisms underlying GM-induced renal injury in addition to the therapeutic and preventative advantages of BV against GM-instigated nephrotoxicity. The underpinning mechanism of the pre-emptive capability of BV against GM-triggered nephrotoxicity is summarized in Figure 9. Although oxidative stress, inflammation, and expression of aquaporins are highlighted in the current investigation, there is limited information on the precise mechanism by which BV functions. Further investigation of more markers is required to validate the efficacy of BV in alleviating GM-induced renal injury in clinical settings. Moreover, future studies incorporating standard antioxidants as positive controls (e.g., N-acetylcysteine or vitamin E) are warranted to benchmark BV’s efficacy against established therapies.

## 5 Conclusion

Overall, GM toxicity resulted in profound kidney injury, attributed to oxidative stress, inflammation, AQP downregulation, and apoptotic cell death. BV could revoke the GM-triggered damage in renal tissues. Its alleviating activity may be attributed to its antioxidant and anti-inflammatory properties. Based on the aforementioned findings, we propose that BV can be deemed an efficient adjuvant to GM therapy in order to mitigate its renal adverse effects via its anti-inflammatory, antioxidant, and antiapoptotic activities and due to its function in the reinstatement of the expression of AQP1 and 2. Future studies are warranted to investigate the efficacy of BV in alleviating GM-induced renal injury in human patients.

## Data Availability

The raw data supporting the conclusions of this article will be made available by the authors, without undue reservation.
