# Supplementary material for: Bee venom ameliorates gentamicin-induced kidney injury by restoring renal aquaporins and enhancing antioxidant and anti-inflammatory activities in rats
Source: Front Pharmacol. 2025 Apr 17;16:1525529. doi: 10.3389/fphar.2025.1525529 (PMC12043641; doi:10.3389/fphar.2025.1525529)
Supplement: Supplementary file 1 [file Table1.docx]

**Table S1. Primers list**

| **Gene** | **Forward (from 5' to 3')** | **Reverse (from 5' to 3')** | **Accession no.** |
| --- | --- | --- | --- |
| P65 | GACGAGGCTCGGAGAGCCCA | CTGGGGCGGCTGACCGAATG | NM_001029913.1 |
| Kim-1 | TATTTGGGGGAACAGGTTGC | CAAGTCACTCTGGTTAGCCGTG | AF035963.1 |
| IL-6 | TCCTACCCCAACTTCCAATGCTC | TTGGATGGTCTTGGTCCTTAGCC | NM_012589.2 |
| TNF-α | AAATGGGCTCCCTCTCATCAGTTC | TCTGCTTGGTGGTTTGCTACGAC | X66539.1 |
| AQP1 | GGCTTCAATTACCCACTGGA | TTGATCCCACAGCCAGTGTA | NM_012778.2 |
| AQP2 | TGTCTC- CTTCCTTCGAGCTG | AGCTCTACAGTCACAGCCTG | NM_012909.3 |
| NRF2 | GCAACTCCAGAAGGAACAGG | GGAATGGCTCTCTGCCAAAAGC | NM_031789 |
| SOD1 | TCTAAGAAACATGGCGGTCC | CAGTTAGCAGGCCAGCAGAT | NM_017050.1 |
| Casp1 | GTGTTGCAGATAATGAGGGC | AAGGTCCTGAGGGCAAAGAG | NM_012762.3 |
| GAPDH | GACATGCCGCCTGGAGAAAC | AGCCCAGGATGCCCTTTAGT | NM_017008 |

**Table S2. Antibodies list**

| **Antibody** | **Manufacturer** | **Catalog no.** | **Clone no.** | **Dilution factor** |
| --- | --- | --- | --- | --- |
| AQP1 | Chongqing Biospes | YPA2098 |  | 1/500 |
| P65 | SANTA CRUZ | Sc-8008 | F-6 | 1/1000 |
| Kim-1 | Chongqing Biospes | YPA2459 |  | 1/1000 |
| P50 | SANTA CRUZ | Sc-8414 | E-10 | 1/1000 |
| NRF2 | SANTA CRUZ | Sc-518036 | H-10 | 1/1000 |
| SOD1 | SANTA CRUZ | Sc-101523 | 24 | 1/1000 |
| AQP2 | Chongqing Biospes | YPA2280 |  | 1/500 |
| TNF-α | Cell signaling | 11948# | D2D4 | 1/1000 |
| Cyt C | Cell signaling | 12963S | 6H2.B4 | 1/1000 |
| IL-6 | Cell signaling | 12912 | D5W4V | 1/1000 |
| Caspase 3 | Cell signaling | #9662 |  | 1/1000 |
| Cleaved-Caspase3 (Asp175) | Cell signaling | #9664 | 5A1E | 1/1000 |
| Β-actin | Sigma-Aldrich | A5441 | AC- 15 | 1/1000 |
| Goat Anti-Rat IgG/HPR | Abcam | ab205720 |  | 1/1000 |
